# Limited thermal plasticity may constrain ecosystem function in a basally heat tolerant tropical telecoprid dung beetle, *Allogymnopleurus thalassinus* (Klug, 1855)

**DOI:** 10.1038/s41598-021-01478-x

**Published:** 2021-11-12

**Authors:** Honest Machekano, Chipo Zidana, Nonofo Gotcha, Casper Nyamukondiwa

**Affiliations:** 1 Department of Biological Sciences and Biotechnology, Botswana International University of Science and Technology, Private Bag 16, Palapye, Botswana; 2 Department of Mathematics and Statistical Sciences, Botswana International University of Science and Technology, Private Bag 16, Palapye, Botswana; 3 Present Address: Department of Zoology and Entomology, University of Pretoria, Private Bag X20, Pretoria, 0028 South Africa

**Keywords:** Ecology, Physiology

## Abstract

Tropical organisms are more vulnerable to climate change and associated heat stress as they live close to their upper thermal limits (UTLs). UTLs do not only vary little across tropical species according to the basal versus plasticity ‘trade-off’ theory but may also be further constrained by low genetic variation. We tested this hypothesis, and its effects on ecosystem function using a diurnally active dung rolling beetle (telecoprid), *Allogymnopleurus thalassinus* (Klug, 1855) that inhabits arid environments. Specifically, (i) we tested basal heat tolerance (critical thermal maxima [CT_max_] and heat knockdown time [HKDT]), and (ii) ecological functioning (dung removal) efficiency following dynamic chronic acclimation temperatures of variable high (VT-H) (28–45 °C) and variable low (VT-L) (28–16 °C). Results showed that *A. thalassinus* had extremely high basal heat tolerance (> 50 °C CT_max_ and high HKDT). Effects of acclimation were significant for heat tolerance, significantly increasing and reducing CT_max_ values for variable temperature high and variable temperature low respectively. Similarly, effects of acclimation on HKDT were significant, with variable temperature high significantly increasing HKDT, while variable temperature low reduced HKDT. Effects of acclimation on ecological traits showed that beetles acclimated to variable high temperatures were ecologically more efficient in their ecosystem function (dung removal) compared to those acclimated at variable low temperatures. *Allogymnopleurus thalassinus* nevertheless, had low acclimation response ratios, signifying limited scope for complete plasticity for UTLs tested here. This result supports the ‘trade-off’ theory, and that observed limited plasticity may unlikely buffer *A. thalassinus* against effects of climate change, and by extension, albeit with caveats to other tropical ecological service providing insect species. This work provides insights on the survival mechanisms of tropical species against heat and provides a framework for the conservation of these natural capital species that inhabit arid environments under rapidly changing environmental climate.

## Introduction

Climate change is expected to increase global mean temperature by 1.5–4.5 °C by the end of the century if mitigation measures fail. In Africa, the past decade has been the warmest on record at 1.78 ± 0.24 ℃^1–5^ with land bordered areas in the dry tropics of Southern Africa experiencing the warmest temperatures at much faster rates than the global average^5,6^. Such rapid increase in temperatures is likely to exceed upper thermal limits (UTLs) for over 40% terrestrial organisms endemic to the region, particularly those providing fundamental ecological functions^7^. Indeed, the evidence for this is manifesting globally^8^ e.g. very recently (June 2021), a record shattering 49.5 ℃ heatwave was recorded in Canada^9^, a scenario that is spatially consistent in other continents (see e.g.,^10–14^). These record-breaking high temperatures are linked to climate change and threaten the survival of insect species responsible for sustaining key ecological functions.

Reports show that, the effects of global warming on ecologically significant arthropods is increasingly becoming apparent across various facets of environmental ecosystems, particularly in the arid tropics^1^. Insect ecologists are thus concerned about the risks of species survival, risks of extinction and associated loss of ecosystem functions. For example, reports suggest that in some parts, the foraging frequency of dung beetles has reduced by 31% in the last quarter of the twentieth century due to climate change associated heat stress^15^. Empirical evidence suggests that the magnitude of heat stress is increasing, threatening the survival of ecologically important insect species^1–3,15^. For example, recent trends show increases in extreme high and low temperatures are more emphasized in the arid tropics^2,3^ likely increasing thermal injury through additive stress, reducing survival, abundance and distribution of dung beetles^16–19^. This has created geographical sub-optimal thermal heterogeneity for tropical insects^17,20,21^ particularly those in hot, arid habitats, likely affecting their organismal function and the efficiency of the whole ecosystem functions. For example, Holley and Andrew^22^ as well as Giannini et al.^23^ showed that heat stress impacts ecological functions in several dung beetle species. For example, in *Onthophagus hectate*, sub-optimal high temperatures reduced both brood ball sizes and depth of burial, likely exposing the next generation immatures to high mortalities due to desiccation and starvation^24,25^.

Heat tolerance is a physiological trait of ecological significance and may determine the fate of tropical organisms in the face of climate change. Insects are more susceptible to snaps of extreme temperature events because their body temperature closely tracks the prevailing ambient environment^26,27^. Tropical organisms are especially vulnerable to heat stress because they live in habitats with temperatures close to their UTLs and often lack the capacity to compensate adaptively through phenotypic plasticity^28–30^ partly due high investment in high basal heat tolerance or genetic constrains^31–33^. Thus, it follows that variation in UTLs is also lower than that of lower thermal limits (LTLs) even across space and species (reviewed in^17^). While UTLs for insects are generally ~ 40 °C with little variation^32^, tropical organisms should thus compensate adaptively in order to maintain functionality under climate change^34^. Unless organisms compensate in situ to heat stress through behaviour e.g. migration to less hostile environments^35,36^, phenotypic plasticity becomes an essential requisite for survival. Therefore, phenotypic plasticity is thus, a critical primary factor in buffering species against the negative effects of heat stress on insect survival through increased magnitudes of thermal safety margins^28,37^ and may help improve fitness under suboptimal conditions. While climate change continues to push tropical insects closer to UTLs^6,28^, those organisms capable of compensating adaptively may emerge winners of climate change^37^. Thus, evidence exists for a strong selection for either high basal heat tolerance and/or plasticity thereof with climate change^38^, depending on species. However, data on specific mechanisms for heat tolerance in individual species of ecological importance is lacking particularly in sub-Saharan dry tropics despite the region’s high vulnerability to warming^5,6^.

The extent to which phenotypic plasticity can buffer climate change effects has been a subject for huge debate^7,28,30,39,40^, and moreso, its effects on the maintenance of essential ecosystem function is unclear^41^. Theories explaining variation in plasticity and basal thermal stress resistance have been equivocal^33^. For example, the ‘trade-off’ hypothesis predicts that higher basal thermal tolerance may come at a cost of phenotypic plasticity^28,42^. This suggests that the scope for tropical organisms to physiologically compensate for UTLs, e.g. critical thermal maxima (CT_max_) is constrained^7,29,43^. Furthermore, the evolutionary potential of UTLs is also limited^31^, and acclimation response ratios for CT_max_ are inherently low^29,44^, likely impacting tropical organisms under high climate change stress. Contrastingly, empirical support for the trade-off theory has been equivocal^34^. For example, while some species trade-off plasticity for high basal heat tolerance^28,29^, some organisms with high basal temperature tolerance are reportedly more plastic too^30,39,45^. This raises significant ecological questions on the fate of individual species success under climate change and warrants more studies to unravel exact relationships between basal stress tolerance and phenotypic plasticity. Tropical diurnal species are constantly exposed to heat stress in their arid, hot habitats during foraging. By constantly being exposed to soil heat, dung beetles should theoretically adapt to heat and presumably have inherent high basal resistance to heat stress. However, it remains unknown which species trade off plasticity for basal high temperature tolerance, and how that is likely to subsequently impact on its essential ecosystem function, e.g. dung removal. Nevertheless, previous reports suggest global warming poses a threat to these species by likely reducing their field fitness and ecological function apart from increasing their risk of extinction^2,24,40,46^.

Dung beetles are coprophagic species that use dung during feeding and nesting^47^. Through their coprophagy, they concomitantly contribute to other ecological functions such as nutrient cycling, secondary seed dispersal, reducing parasites and the loss of N_2_ due to ammonia volatilization^48–53^. They also contribute to reduction in greenhouse gas emissions and facilitate microbial activity through bioturbation^54,55^. Thus, dung removal is a valuable economic contribution to functional efficiency used as a proxy for ecological function provision by dung beetle species^22,25,56^. This makes dung beetles a critical resource (natural capital) globally. The coprophagic teleocoprid, *Allogymnopleurus thalassinus* is a significant ecological function contributor in southern Africa where it is native^57^ although it is widely distributed in other hot arid environments throughout the continent (^57,58^ (online database)). Its local abundance makes it a significant component of local biodiversity assemblages, community structure and natural capital^57,59,60^. Given the current and projected increase in mean temperatures of the savannah land mass with climate change^1,3^, it remains unknown how this important species may survive heat stress and whether or not it can remodel its thermal phenotypes through plasticity (see e.g.,^39,42^). Although several studies have assessed physiological responses of insects to climate change before (see^7,17^), only a few have considered the combined effects of physiological and ecological impacts of thermal stress^61^. Similarly, while the effects of temperature on dung beetle functional responses are documented^24,25,50,61–63^, to our knowledge, no studies have simultaneously assessed acclimation effects on both physiological and ecological responses of dung beetle species in sub-Saharan Africa (see e.g.,^64,65^). Furthermore, if indeed *A. thalassinus* is vulnerable to heat stress, the fate of its ecological function associated with heat stress is largely unknown. Building on latitudinal hypothesis^66^ and the findings by van Heerwaarden et al.^29^, Gunderson and Stillman^28^ and van Heerwaarden and Kellermann^40^, we hypothesise that (i) *A. thalassinus* may have high basal heat tolerance as a native tropical day active forager and that (ii) if present, this high basal heat tolerance is likely traded-off with phenotypic plasticity (see also^28,67,68^). Here we used ecologically relevant physiological—(CT_max_ and heat knockdown time [HKDT]) and ecological-traits as proxies for performance under climate change^46,69^ using dynamic protocols^64^. The results will provide a profound understanding of species-specific physiological acclimation capacity and associated ecological implications for this ecologically important species. This information is essential to predict the fate of ecosystem function under climate change^70^ and provide a framework for the conservation of such species in order to preserve their benefits to the environment and society in the future.

## Results

### Basal critical thermal maxima and heat knockdown time

**Figure 1:**
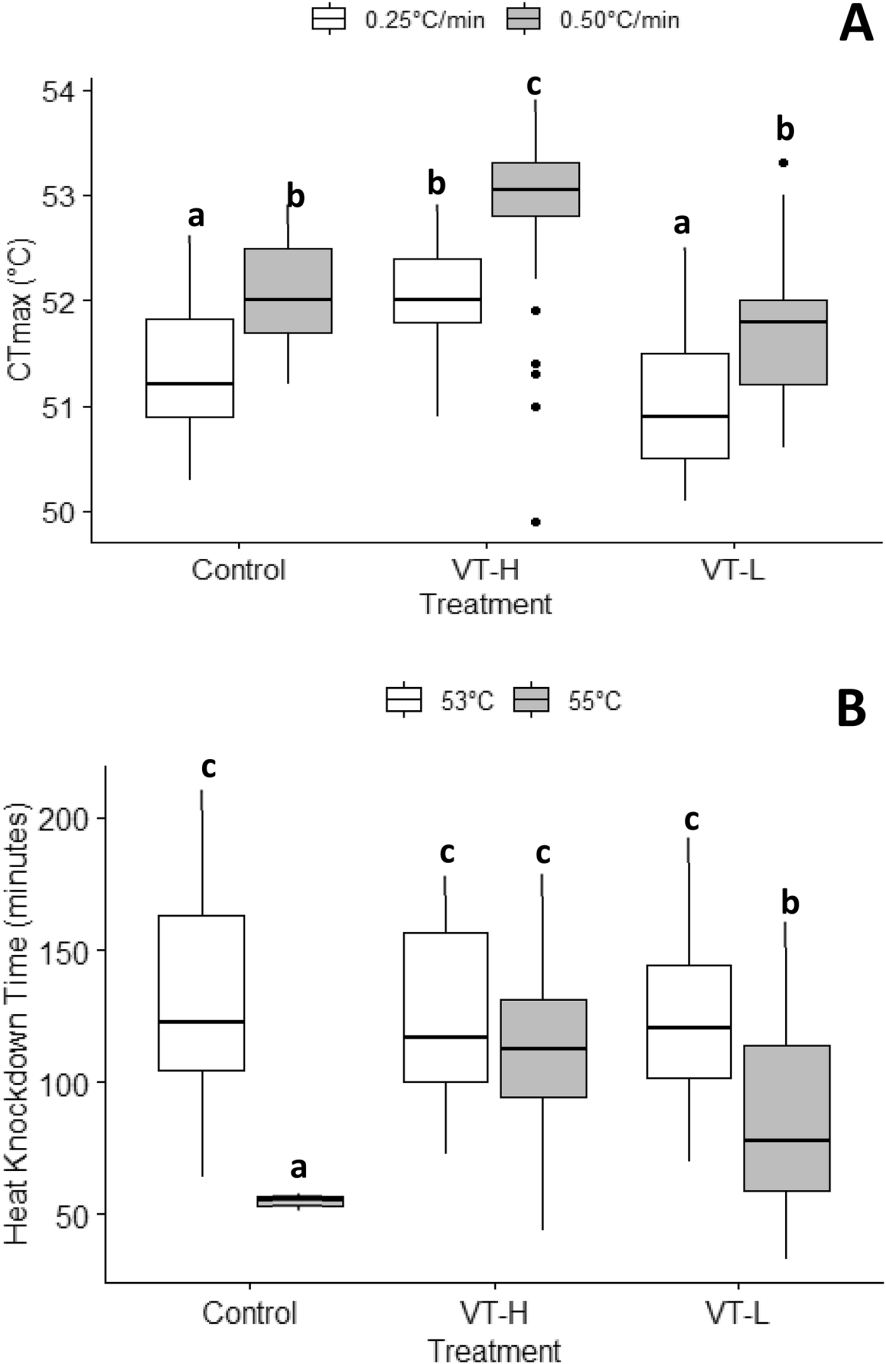
The effect of ramping rate and variable temperature acclimation on (**A**) critical thermal maxima (CT_max_) and, (**B**) heat knockdown time (HKDT) at 53 °C and 55 °C. Each point represents mean ± SEM and median ± SE for (**A**) and (**B**) respectively.

The CT_max_ of *A. thalassinus* was higher than 50 °C for all treatments and controls (Fig. 1A; Table 1). The CT_max_ of non-acclimated *A. thalassinus* beetles was 51.32 ± 0.68 °C and 51.73 ± 1.81 °C, at ramping rate of 0.25° and 0.5 °C/min respectively. These CT_max_ values indicate extreme heat tolerance and are comparable with other related diurnal beetle species in the same tribe (Table 1). Similarly, basal HKDT was long, recording 130.79 ± 41.17 and 55.05 ± 2.12 min at 53 and 55 °C respectively. These HKDT values are also longer that related diurnal species in the same tribe (Table 1).

**Table 1:** The basal CT_max_ (°C) and HKDT (minutes) for *Allogymnopleurus thalassinus* compared to other diurnal telecoprid species in the same tribe occurring in the same hot and arid environment.

Tribe	Species	CT_max_ (°C) (± SEM)	HKDT (minutes) (± SE)	References
Gymnopleurini	*Allogymnopleurus thalassinus*	51.3 ± 0.68	130.8 ± 41.12	Current study
Gymnopleurini	*Gymnopleurus aenescens*	52.5 ± 0.15	19.6 ± 1.12	^71^
Gymnopleurini	*Gymnopeurus ignitus*	52.1 ± 0.23	17.7 ± 1.04	^71^
Oniticellini	*Euoniticellus intermedius*	48.9 ± 0.14	57.8 ± 1.62	^71^

### Effect of acclimation on heat tolerance

CT_max_ was significantly affected by both acclimation treatments (*p* < 0.001) and ramping rate (*p* < 0.001) although combined interactions of acclimation and ramping rate were not statistically significant (*p* = 0.924) (Table 2). Variable high temperature acclimation (VT-H) significantly improved CT_max_ compared to control (*p* < 0.001) at all the two ramping rates while variable low temperature (VT-L) on the other hand significantly reduced CT_max_ (*p* = 0.0274) compared to the controls (Fig. 1A). Across all acclimation treatments, the 0.5 °C/min ramping rate resulted in significantly higher CT_max_ values than the 0.25 °C/min one (Fig. 1A).

**Table 2:** The effect of acclimation treatment and ramping rate on CT_max_.

Parameter	DF	SS	SM	F	Pr(< F)
Treatment	2	32.76	16.38	36.906	< 0.001
Ramping rate	1	24.58	24.58	55.389	< 0.001
Treatment × ramping rate	2	0.07	0.035	0.079	0.924
Residuals		171	75.89		0.444

DF = degrees of freedom, SS = sum of squares, SM = mean sum of squares, F = F ratio, Pr(< F) = *p* value associated with the F-statistic.

HKDT was significantly affected by both acclimation treatments and knockdown temperature (*p* < 0.05), with combined interactions of both acclimation treatments and knockdown temperature also being statistically significant (*p* < 0.05) (Table 3). The HKDT for control adult beetles was 130.79 ± 41.17 and 55.05 ± 2.11 min for knockdown temperature of 53 and 55 °C respectively. HKDT was consistently significantly higher across all treatments for the 53 °C than 55 °C heat knockdown temperature (Table 3). Variable temperature high and low acclimation treatments improved HKDT for the 55 °C heat knockdown temperature but not 53 °C. Therefore, there were no treatment effects for HKDT at the 53 °C heat knockdown temperature. Interactions between acclimation treatment × heat knockdown temperature showed that, at 53 °C the HKDT for both acclimation treatments (VT-H & VT-L) were not statistically different from the control group, while at 55 °C, both variable temperature high and low acclimation treatments significantly increased HKDT (Fig. 1B).

**Table 3:** The effect of acclimation treatment and ramping rate on HKDT.

Parameter	F	DF	DF-Res	Pr(< F)
Treatment	10.415	2	145	< 0.001
HK*T*	94.211	1	145	< 0.001
Treatment × HK*T*	13.242	2	145	< 0.001

DF = degrees of freedom, DF-Res = degrees of freedom residuals, F = F ratio, Pr(< F) = *p* value associated with the F-statistic.

### Effects of acclimation on ecological functions

**Figure 2:**
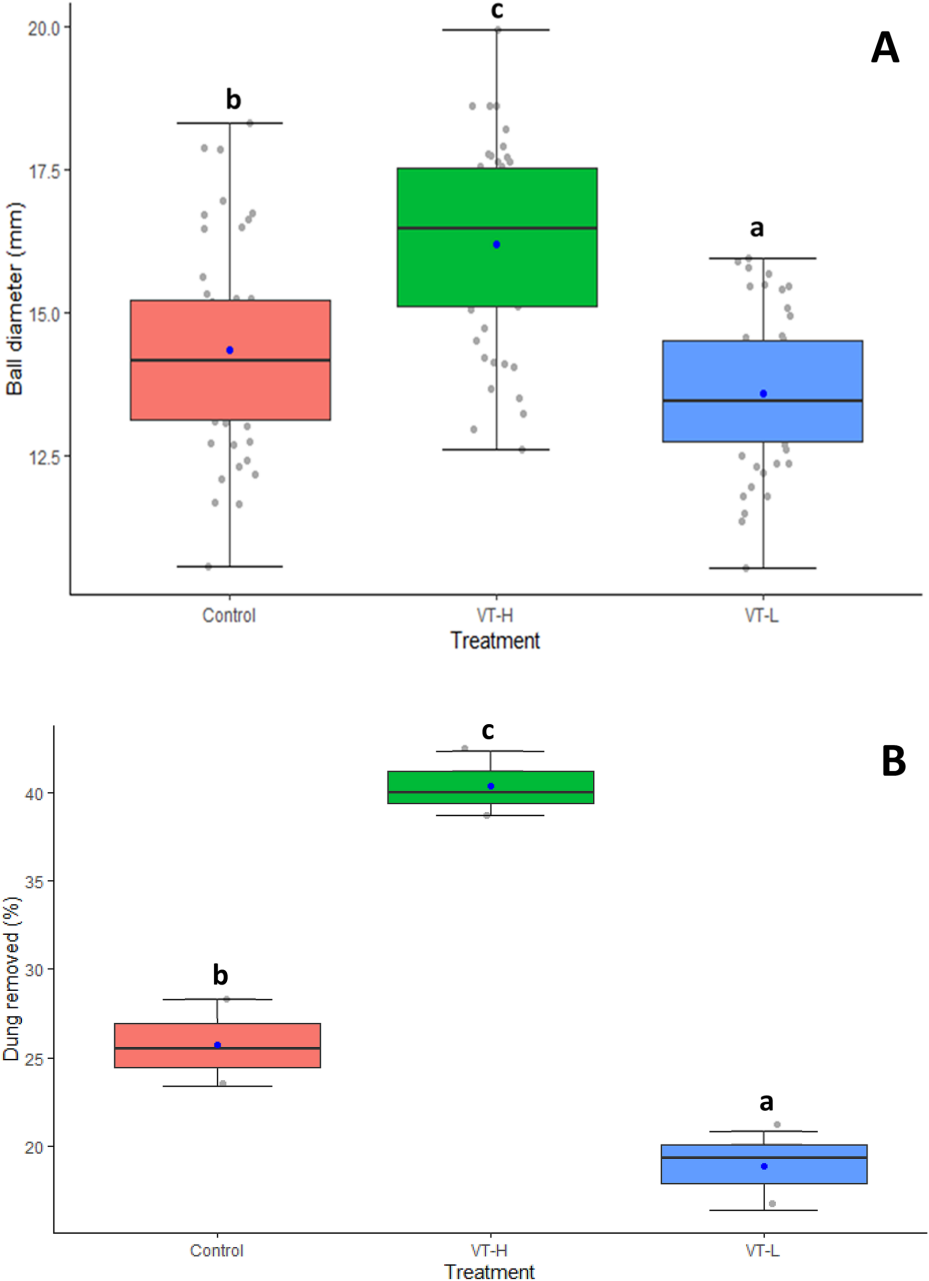
The effect of acclimation treatments on (**A**) ball diameter, and (**B**) dung removal efficiency. Each point represents mean ± SEM.

Acclimation treatments significantly affected the mean ball diameter made by *A. thalassinus* (F_(3, 147)_ = 4376.8, *p* < 0.001). Variable temperature high acclimated beetles made significantly wider balls (16.19 ± 1.30 mm; *p* < 0.001), than both the non-acclimated beetles (14.35 ± 1.31 mm; *p* < 0.001) and variable temperature low treatments (13.58 ± 1.14 mm; *p* < 0.00001) (Table 4; Fig. 2A). Similarly, both acclimation treatments had significant effects on the proportion of dung removed (F_(3, 6)_ = 554.32; *p* < 0.001). The proportion of dung removed followed the same trend; where variable temperature high acclimated beetles removed a significantly higher proportion of dung compared to controls (Table 4; Fig. 2B). Furthermore, variable low temperature acclimation came at an ecosystem function cost, significantly reducing dung removal compared to controls (Table 4; Fig. 2B).

**Table 4:** Parameter estimations of variable high (VT-H) and variable low (VT-L) acclimation treatments on ball diameter and dung removal efficiency.

Trait	Treatment	Estimate (mean)	SE	t-value	Pr( >|t|)
Ball diameter	Control	14.3463	0.2229	64.37	< 0.001
	VT-H	16.1863	0.2229	72.63	< 0.001
	VT-L	13.5778	0.2229	60.92	< 0.001
	Control	25.753	1.261	20.42	< 0.001
Dung removal	VT-H	40.323	1.261	31.98	< 0.001
	VT-L	18.853	1.261	14.95	< 0.001

Statistical significance was determined at *p* < 0.05.

### Acclimation response ratio (ARR) of CT_max_

The acclimation response ratio (ARR) of CT_max_ at benign ramping rate (0.25 °C/min) for *A. thalassinus* following variable temperature high and variable temperature low acclimation treatments was 0.0284 °C/°C and − 0.02556 °C/°C respectively, indicating ~ 2.84% compensation capacity and ~ − 2.56% fitness cost respectively. However, when the CT_max_ ramping rate was increased to 0.5 °C/min, the CT_max_ ARR for variable temperature high acclimation treatment increased to ~ 0.06 °C/°C, (i.e. 6% compensation capacity) while it decreased for variable temperature low (− 0.026 °C/°C) (-2.6% fitness cost). Compared to results of ARR values of other insects’ taxa at a chosen ramping rate of 0.25 °C/min, the CT_max_ ARR values for *A. thalassinus* was low except compared to *Scarabaeus zambezianus* and *Copris elephenor* (Table5).

**Table 5:** The critical thermal maxima (CT_max_) acclimation response ratios (ARR) for *A. thalassinus* compared to other different Orders and their specific species obtained from literature.

Order	Species	CT_max_ ARR (°C/°C)*	References
Coleoptera	*Allogymnopleurus thalassinus*	0.0284	Current study
	*Metacatharsius opacus*	0.085	^72^
	*Scarabaeus zambezianus*	− 0.030	^72^
	*Copris elephenor*	− 0.420	^72^
	*Linepithema humile*	0.20	^73^
	*Castanophlebia calida*	0.19	^74^
Hymenoptera	*Lestagella penicillata*	0.16	^74^
Ephemeroptera	*Drosophila melanogaster*	0.043	^75^
	*Portunus pelagicus*	0.280	^76^
Diptera	*Scylla olivacea*	0.495	^77^
Decapoda	*Thalameta crenata*	0.360	^77^

The list may not be purely exhaustive but represents a significant number of studies found in literature at the time of publication. Also note that CT_max_ values and consequently ARRs may vary depending on CT_max_ methodological context e.g. starting temperature^17^. All the acclamatory response ratio values were calculated only for CT_max_ at ramping rate of 0.25 °C/min.

## Discussion

Our results showed that the native, arid environment inhabiting dung beetle *A. thalassinus* has high basal heat tolerance as exhibited by CT_max_ values > 50 °C and long HKDT values (> 2 h), consistent with other arid/desert habitat insect species that exhibit these striking basal heat tolerance traits^18,40,43,78,79^. Second, variable temperature high acclimation improved CT_max_ for all ramping rates, while variable temperature low acclimation generally reduced heat tolerance (CT_max_) This may show plastic responses to CT_max_ at high temperature acclimation. Similarly, heat knockdown temperature influenced HKDT, suggesting that the magnitude of temperature stress may affect insect fitness in a warming climate (see^40^). Similar, to CT_max_, effects of acclimation on HKDT were significant, with variable temperature high and low temperature acclimations both increasing HKDT than controls at 55 °C while no acclimation treatment effects were recorded for 53 °C. The improved CT_max_ at higher ramping rate is in agreement with the notion that thermal stress and injury is highly additive^19^. Thirdly, high temperature acclimation increased net ecological function through significantly higher dung removal and wider ball diameters than low temperature acclimation. Fourth, a qualitative comparison of ARRs to like tropical insect species showed that phenotypic plasticity of heat tolerance in *A. thalassinus* adults was low, indicating that plasticity is constrained^28,37^. When *A. thalassinus* ARR was compared to the general mean ARR for most terrestrial arthropods (0.12–0.16)^28^ its ARR was ~ 5 times lower, indicating considerably constrained high temperature plasticity compared to other insect species. Thus, this work supports the trade-off theory^28,29,40,67^, and that selection for high basal heat tolerance in *A. thalassinus* may come at a cost of plasticity. This therefore affirms the notion that although having high basal heat tolerance than temperate species, tropical species may be more vulnerable to future global warming because they lack complete plastic responses to heat stress, i.e. phenotypic plasticity is ecologically insufficient (^29^, but see^36^). Acclimation effects were significantly positive on ecosystem function only following high temperature acclimation and would likely plateau. We therefore summarise that although plasticity is present in *A. thalassinus*, it may be insufficient to buffer this species against projected increase in in global warming, moreso in its arid, tropical native habitat of southern Africa. Thus, *A. thalassinus’* field fitness and efficiency in contributing to ecological functions in the future may also be constrained under increasing heat stress with climate change.

Critical thermal maximum is a popular ecologically relevant index of heat tolerance measurement^18^ that is used by many ecologists to measure the capacity of organisms to survive extreme heat and thus as proxy for estimating climate change risks in many species^40,69,72,75^. Our results showed that regardless of acclimation and ramping rates, the basal CT_max_ for *A. thalassinus* was high (> 50 °C), indicating that *A. thalassinus* is thermophilic (survives extreme temperatures of > 41 °C)^17^. This could be attributed to its tropical origins^57,59^ and day foraging activities^61,71^ in tropical environments where environmental temperatures are normally high during daytime. Similarly, in seed bugs, Käfer et al.^79^ showed high correlation between CT_max_ with environmental annual mean temperature and mean maximum temperature of warmest months in Austria. In separate studies, the desert ant, *Cataglyphis bombycin* had an extremely high heat tolerance (CT_max_ = 53.6 °C) (reviewed in^17,80^), partly attributable to its high habitat temperature (desert) environment. Similar reports on dung beetles *Gymnopleurus aenescens* and *Gymnopleurus ignitus* from the same habitats (Khumaga, Botswana) reported CT_max_ values ranging 52–53 °C^71^, further confirming how environment^79^ may shape basal heat tolerance and adaptation thereof in insects.

HKDT for *A. thalassius* was long (> 2 h), at 53 °C HKT, again attesting its high heat tolerance apart from high CT_max_. This result is in tandem with Gotcha et al.^71^, who showed high HKDT in related diurnal dung beetles *Gymnopleurus aenescens* and *Gymnopleurus ignitus*. Similar observations were observed by Nyamukondiwa et al.^72^, using dynamic acclimation protocols for nocturnally active dung beetle *Scarabaeus zambezianus*. This high basal HKDT (and CT_max_) in *A. thalassinus* could have evolved as an adaptation to daytime activity (foraging during peak heat stress) in stressful arid environments^71,79,81^ where it is native^57^. Although diurnal species are highly heat tolerant physiologically, telecoprids were observed to particularly employ behavioural plasticity mechanisms such as utilising microhabitats (see details in^35,36^) or using the moist dung balls as thermal refuge (heat sinks) to cool their bodies during rolling^82^. Although it is not clear under what environmental temperature this thermal respite behaviour would be initiated, in the midday foraging desert ant, *Ocymyrmex robustior*, thermal respite behaviour was shown to increase when soil temperatures reach 51 °C, coinciding with *A. thalassius* CT_max_. Thus, extreme basal heat tolerance for *A. thalassius* reported, here may be attributable to its tropical origins^57^ and the latitudinal hypothesis^66,79^ and may form part of its main survival strategy against warming climates.

Our results showed that variable temperature high acclimation improved CT_max_, while variable temperature low acclimation generally reduced CT_max_. This result suggests plastic responses for this species for CT_max_ following dynamic high temperature acclimation. These results are consistent with results from other studies which showed that heat acclimation improves CT_max_, while on the contrary, low temperature acclimation may not improve heat tolerance^17^. Similar to what was observed in other studies, CT_max_ values increased with increase in ramping rate, while slower ramping rates reduced this trait potentially owing to cumulative stress effects^17,19^. The effects of ramping rates, test temperatures and duration of acclimation are reportedly complex to disentangle^17,46^. Indeed, CT_max_ varies with methodological context e.g., starting temperature and ramping rates^83,84^. Thus, the higher plasticity (CT_max_) and survival consequences for higher ramping rates (for CT_max_) may be attributable to the effect of reduced timing (and stress thereof) at faster heating rates (0.5 °C/min) relative to slower one (0.25 °C/min). This is in keeping with recent models that assume heat stress and consequent injury is a function of temperature severity and that it is additive^19^.

Acclimation to high temperature often improves low temperature traits and vice versa owing to shared physiological response mechanisms e.g., Hsps^85^. However, phenotypic plasticity may also be maladaptive and traded-off with other life history traits^86^. In related studies, Kristensen et al.^87^ showed that acclimation to low temperature negatively affected heat tolerance in *Drosophila melanogaster*. This suggests a possible trade-off between heat and cold tolerance, as such, represents an additional constraint for this species when facing changing environments (i.e. acute high and low temperature events) in nature. This also affirms the notion that the relationship between heat and cold shock responses is highly asymmetrical e.g., heat acclimation ‘always’ improves low temperature survival while the reverse is not always true (see discussions in^17^). Similarly, in *Nezara viridula*, CT_max_ showed more plastic responses post heat acclimation than CT_min_, showing that CTLs may be typically decoupled^88^. In wolf spiders, acclimation also did not modify thermal breadth showing that low thermal plasticity, as reported for *A. thalassinus* here, may not cushion these species from high temperature stress^89^. Thus, the role of short- to medium-term plasticity in the adaptation to variable climatic environments remains largely contested^30^.

One of the more intriguing aspects of our data is the implications of variable temperature acclimation on *A. thalassius* functional responses (dung removal efficiency). High temperature acclimated adult beetles made significantly bigger balls and removed a significantly higher proportion of dung compared to both control and low temperature acclimated beetles. In our view, this translates to relatively higher ecological functions at high temperature acclimation compared to the control and low temperature acclimated beetles. In a similar study, Mamantov and Sheldon^25^ showed that *Onthophagus taurus* increased ball size and depth of dung burial following high temperature acclimation, signifying that ecological responses were linked to temperature acclimation. During acclimation, higher temperatures increase metabolic enzyme activity^90^ that likely plateaus at peak (yet unknown) temperature or duration of exposure.

Our results also showed that low temperature acclimation had negative effects on dung beetle ecosystem function, manifesting as (significantly smaller dung balls, and lower dung mass removal). Acclimation responses to low temperature may be highly species dependant^61^; as such, we speculate, with caveats that *A. thalassius* may not be adapted to low but high temperature stress owing to its warm tropical origin and diurnal activity patterns (see details in^61^). In a similar study, Wu and Sun^24^, showed that a 2.3 °C increase in temperature delayed oviposition maturity and egg hatching by 4.1 and 7.2 days respectively and egg and larval size by 22.1 and 33.4% respectively in *Aphodius erractus*. This signifies that high temperature acclimation affects beetle life history fitness traits. The current study only tested within-generation adult acclimation responses; thus, future studies should aim to investigate the effects of temperature variability across generations and testing more diverse life history traits. In addition, we could not account for the cost of mounting plasticity and the role of behavioural adaptation (the Borget Effect)^25,28,40,74^. Thus, future work may need to test acclimation across ontogeny and investigate the role of carry-over and/or transgenerational plasticity in *A. thalassius*. To avoid competition, dung rollers are also known to abandon their dung balls if they cannot migrate far enough from the dung pat source. As such, future experiments may consider increasing experimental arena sizes, considering mesocosm- or field-approaches to better explain the effects of temperature variability on ecological services. Similarly, future studies may also incorporate dung beetles from diverse locations to better understand the role of local adaptation in buffering climate change effects.

Although critical thermal limits (e.g., CT_max_) have received considerable attention under climate change, the fate of ecosystem functioning under climate change have been limited (but see^22,61,91^). Our work thus, provide novel data on how a diurnally active tropical dung beetles species may be adapted to the predicted global warming and how the ecological service delivery of this species may be affected by heat stress under climate change. This is evidence to argue that future models for species survival under climate change should account for potential losses in ecosystem function and/services. Our results showed that (i) *A. thalassius* has extreme basal heat tolerance that presumably helps the species forage diurnally in heat stressing tropical environments; (ii) acclimation to variable high temperature improves *A. thalassius* heat tolerance indicating thermal plasticity (albeit limited), while simultaneously improving ecosystem function (dung removal), (iii) low temperature acclimation constrained *A. thalassius* ecosystem function (dung removal), and (iv) *A. thalassius* has low plasticity as exhibited by the low ARRs, as such phenotypic plasticity may unlikely cushion physiological fitness and survival of this species and indeed ecosystem functioning in the face of heat stress associated with climate change. Our study thus contributes empirical evidence to literature supporting the phenotypic plasticity versus basal tolerance ‘trade-off’ theory and contributes to the growing recognition of the need to make practical decisions for ecosystem management to enable continued provision of ecological functions under a range of future human-mediated environmental conditions in sub-Saharan Africa and similar environments.

## Materials and methods

### Study animals

Study beetles were collected from Khumaga Village (S20.46801; E24. 51491; 918 m.a.s.l), Central District, Botswana, in February 2020. The summer season represents the peak activity time of most dung beetles. Khumaga village is characterised by small scale pastoralism (mainly cattle and goats) and is at the interface with a protected area (Wildlife Park), Makgadikgadi Pans National Park, that hosts several wild large ruminants, and non-ruminants^92^. This rich animal diversity provides diverse and overlapping dung resources that promote abundant and diverse beetle communities. The beetles were captured using pit fall traps consisting of mini-plastic buckets (~ 2 L) buried flush with the ground and covered with fine wire mesh of 15 mm internal diameter (modified from^71,72,93,94^). About 350 g of fresh cattle dung was placed on top of the wire mesh as bait. The traps were covered with overhead shading to protect from rain and direct sunlight^94^. Traps were set at 06:00 h every morning and captured beetles were collected from about 1000 h till 1800 h for 6 consecutive days. Collected beetles were placed in insulated cooler boxes with perforated lids containing moist soil and dung for feeding during transportation to the Eco-physiology Laboratory, Department of Biological Sciences and Biotechnology, Botswana International University of Science and Technology in Botswana. In the laboratory, beetles were identified using gross morphology^95^ and Voucher specimens were deposited at the Botswana National Museum. Beetles were kept in a climate chamber set at conditions like those at site of collection (28 ± 1 °C, 65 ± 10% RH, 14L:10D photoperiod) (see^72^) prior to experimentation. All acclimations and/or experiments were done within 7 days (as in^96^) of specimen collection to minimise confounding effects of laboratory captivity.

### Thermal variability acclimation treatments

**Figure 3:**
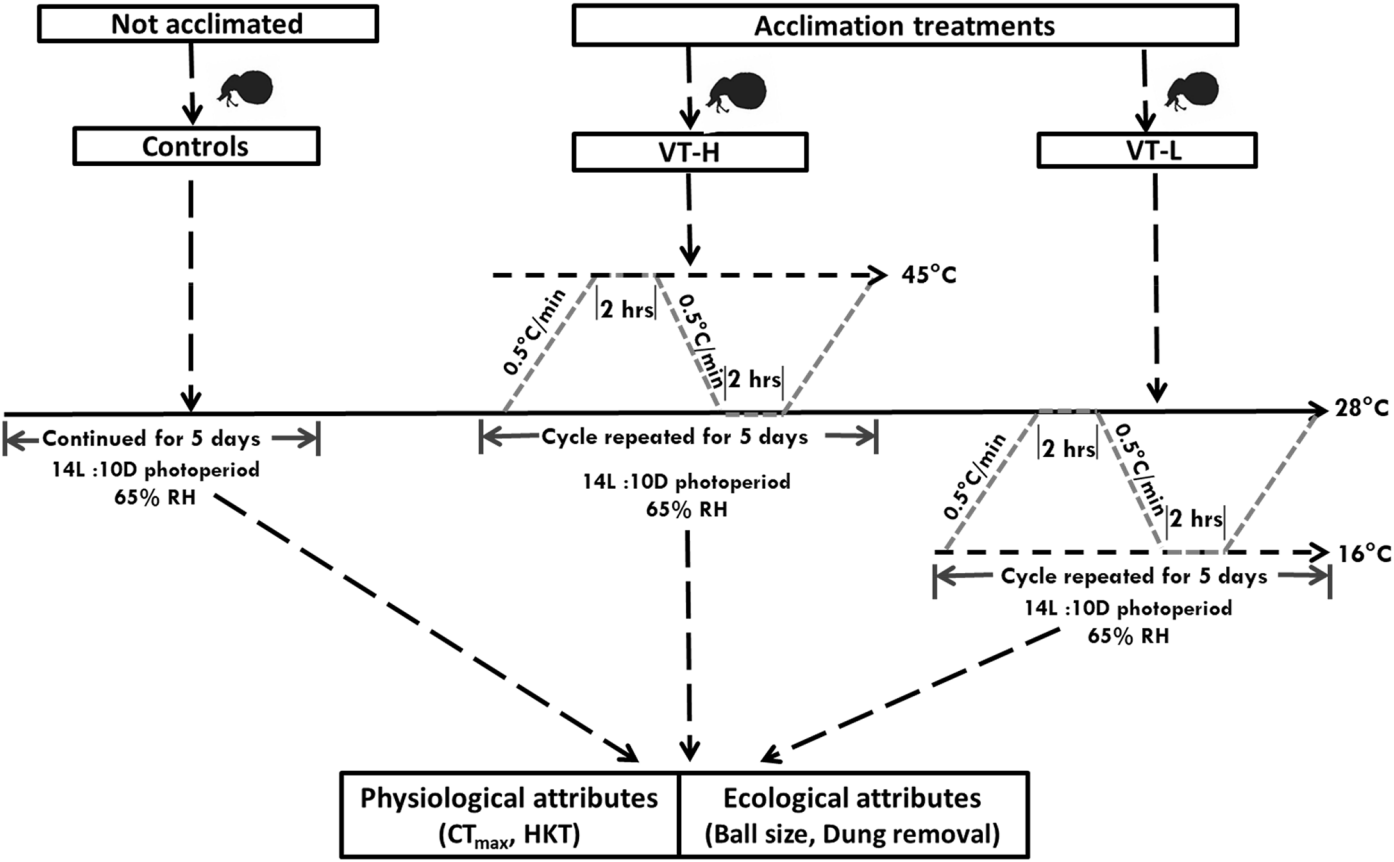
Schematic representation of variable temperature acclimation treatments at a ramping rate of 0.5 °C/min from a benign temperature of 28–45 °C (VT-H) and to 28–16 °C (VT-L) at 65 ± 10% RH and 14D:10L photoperiod. Control beetles were maintained at a constant 28 °C, 65 ± 10% RH and 14D:10L photoperiod.

Beetles were acclimated using a combination dynamic (fluctuating temperature) protocol in climate chambers (HPP 260, Memmert GmbH + Co.KG, Germany) at 65 ± 10% relative humidity (RH) under 14L:10D photoperiod. This dynamic acclimation protocol is ecologically sound and may give more reliable estimates of species responses to variability typical of environmental climate change^97,98^. For variable high temperature acclimation (VT-H), temperature was ramped up at 0.5 °C/min from a benign (optimum) temperature of 28–45 °C, allowed to remain at 45 °C for a duration of 2 h before being ramped down back to 28 °C, remain constant for 2 h (at 28 °C) before ramping up again in continuous repeated dailycycles (Fig. 3). Similarly, for variable low temperature acclimation (VT-L), temperature was ramped down at 0.5 °C/min from a benign of 28 °C (ambient) to 16 °C, and then held at 16 °C (2 h) before being ramped up back to 28 °C, held there for 2 h before ramping back down in repeated daily cycles (see Fig. 3). Acclimation at both temperature extremes may improve both high and low temperature tolerance^85,99^ owing to potential overlap in heat and cold stress resistance mechanisms. Control beetles were maintained at a constant 28 °C. Relative humidity and photoperiod were maintained at 65 ± 10% (RH) and 14D:10L respectively for all treatments (see also^72^).

### Physiological assays

To investigate the effects of plasticity on physiological fitness, we measured physiological functional traits *vis* CT_max_ and HKDT in adults following standardised protocols from^72^. Critical thermal maximum is a good indicator for an organism's ability to survive extreme events, and as such a good measure of resistance mechanism under extreme heat exposures^100^. A series of insulated double-jacketed chambers (‘organ pipes’) was connected to a programmable water bath (Lauda Eco Gold, Lauda DR.R. Wobser GMBH and Co. KG, Germany) filled with 1:1 water:propylene glycol to allow for sub-zero temperatures at the same time regulating the flow of liquid around the chambers. Ten mixed sex adult beetles were counted and individually placed randomly into the organ pipes. In the organ pipes, beetles were allowed to first equilibrate for 10 min at 28 °C (benign temperature), before ramping waterbath temperature up at a benign rate of 0.25 °C/min (see^69^) until the CT_max_ for each beetle was recorded. Thermal ramping rates may affect adaptive capacity for UTLs^64,69^. Thus, the process was repeated using a faster ramping rate of 0.5 °C/min with a fresh set of beetles. A thermocouple (type K 36 SWG) connected to a digital thermometer (53/54IIB, Fluke Cooperation, USA) was inserted into a control chamber to measure beetle temperature. The body temperature of each individual beetle was assumed to be in equilibrium with the organ pipe temperature as in similar work (see^72^). Each beetle was discarded after recording and for each ramping rate, the process was repeated three times with fresh beetles each time to yield sample size of n = 30 (30 replications) for each treatment. In this study, CT_max_ was defined as the temperature at which each individual beetle lost coordinated muscle function, consequently losing the ability to respond to mild stimuli like prodding with thermally inert camel-hair brush (e.g.^72,101^).

For HKDT, ten mixed sex beetles were individually placed in numbered 30 ml polypropylene vials and placed in a climate chamber set at 53 ± 0.5 °C (65 ± 10% RH) connected to a camera (HD Covert Network Camera, DS-2CD6412FWD-20, Hikvision Digital Technology Co., Ltd, China) linked to a computer from where observations were recorded (in minutes). The process was repeated at 55 ± 0.5 °C (65 ± 10% RH) and each experiment was run three times with fresh beetles each time to yield a sample size of n = 30 (30 replications) for each acclimation treatment and each HKDT temperature. The HKTs were selected following both preliminary assays and previous studies^71^. Beetles were discarded after each recording. HKT was defined as the time (in minutes) at which each individual beetle lost coordinated activity due to acute heat stress (see^72,102^). All treatments and replicates were all randomised across the different experimental blocks.

### Ecological functions

To investigate ecological effects of acclimation, we assessed the effects of treatments on two essential ecosystem functions, dung ball size and dung removal. Ball sizes were measured for each of the three treatments following modifications of methods by^103,104^. Fifty mixed sex beetles from each treatment were provided with 500 g of manually homogenised fresh cattle dung^54^ in plastic containers of 4.09 L total volume, with effective soil depth of 6 cm. The experiment was replicated 3 times for each of the treatments (VT-H, VT-L and controls). Experimental containers were placed in a climate chamber (HPP 260, Memmert GmbH + Co.KG, Germany) set at 28 °C and 65 ± 10% RH under 14L:10D. After 24 h, 50 completely formed balls (see^103^) were randomly picked from each container and ball sizes (diameter) were recorded. Ball diameter was measured using an electronic digital Vanier calliper (E-base Measuring Tools Co., model: SV-03-150, size 6 in./150 mm, Pert Industries, Johannesburg, South Africa). Since most balls were more spheroidal in shape, both the longest and shortest diameters of each ball were measured. The final diameter of each ball was thus calculated as the average of the longest and the shortest diameters.

Dung removal experiments were conducted following modified protocols^54,104,105^. Following acclimation treatments, 50 mixed sex beetles were exposed to 200 g homogenised dung pats (RADWAG1 Wagi Elektroczne, Model AS220. R2, Poland) in plastic containers of 27 × 17.8 × 8.5 cm (4.09 L volume) with effective soil depth of 6 cm. A thin film of clean multipurpose wiping paper was placed beneath each dung pat to avoid soil sticking to the dung. The experiment was replicated 3 times for each of the treatments. Experimental containers were placed in a climate chamber (HPP 260, Memmert GmbH + Co.KG, Germany) set at 28 °C and 65 ± 10% RH under 14L:10D photoperiod. After 24 h, residual dung that was not balled or buried was weighed and recorded. Water loss was accounted for by using a parallel control experiment with only 200 g dung pats but no beetles^93^.

### Data analysis

All the statistical analyses were performed in R version R4.0.2^106^. We built models based on how treatments (acclimation and ramping rates) affected traits of CT_max_ (°C)_,_ HKDT (minutes), ball sizes (mm) and dung removal (%). A total of 180 observations were used to assess the effects of the 3 acclimation treatments (Control, VT-H and VT-L) and ramping rate (0.25 and 0.50 °C/min) on CT_max_.

A sample of 180 observations was also considered for HKDT model at three different acclimation treatments (Control, VT-H and VT-L) with 2 different heat knockdown temperature levels (53 °C and 55 °C) within each treatment respectively. A balanced 90 observations for each of the temperature levels was used treating the data at 53 °C and 55 °C separately for analysis. A preliminary two-way ANOVA on both CT_max_ and HKDT models were run and showed that the residuals were not normally distributed for HKDT. Thus, a rank based non-parametric approach was adopted to assess the effects of acclimation on HKDT under the different acclimation treatments, and heat knockdown temperatures. We used the aligned rank transformation (ART) for nonparametric factorial analyses using only ANOVA procedures^107^. A multifactor contracts procedure by^108^ was implemented to distinguish significant differences on the different factors and levels for the ART method. In order to determine how ball size and dung removal were influenced by the different acclimation treatment levels, one-way analysis of variance (ANOVA) was used. The models were both appropriate as both Shapiro Wilk’s test for normality and Levene’s test for equal variance assumptions on residuals were satisfied. Shapiro Wilk’s *p* values were (0.85, 0.34) whilst Levene’s test *p* values were (0.17, 0.93) for the ball diameter and dung model, respectively.

The responses of *A. thalassinus* to acclimation, termed acclimation response ratio (ARR) was calculated for CT_max_ using the formula:

$$ARR=\frac{{\Delta CT}_{max}}{\Delta Acclimation}$$

where ΔCT_max_ = recorded change in (CT_max_) (°C), and ΔAcclimation = Difference between holding and acclimation temperature (°C) following methods by^44,76,77^. This was compared to ARRs from literature to interpret how the magnitude of plasticity of thermal tolerance may likely buffer *A. thalassinus* under changing climates. Acclimation Response Ratio of 1 shows a positive 1 °C shift in CT_max_ for each 1 °C acclimation temperature investment suggesting positive plasticity while an ARR of close to zero indicates lack of plasticity and ARR = 0.5 indicates little effects of acclimation on CT_max_ plasticity^100^.
